# City economies and microbusiness growth

**DOI:** 10.1177/0042098016680520

**Published:** 2017-01-11

**Authors:** Donald Houston, Darja Reuschke

**Affiliations:** University of Portsmouth, UK; University of Southampton, UK

**Keywords:** agglomeration, growth, home-based businesses (HBBs), microbusinesses, microenterprises

## Abstract

In developed countries, microbusinesses (those employing fewer than 10 people) and home-based businesses have been systematically overlooked in urban economic development thinking. This article assesses the influence of city location and being run from the business owner’s home on microbusiness growth, based on empirical analysis of panel firm-level data over a four-year period during the UK’s long boom. The analysis reveals that cities provide benefits to microbusinesses for turnover growth but not for employment growth – suggesting that the additional growth induced by cities for microbusinesses may be jobless growth. However, in the case of microbusinesses run from the owner’s home, cities facilitate growth into medium-sized businesses (with 50+ staff). In conclusion, microbusinesses, including those run from business owners’ homes, are integral to the evolution and dynamics of urban economies and essential to understanding the nature of growth in cities. Agglomeration theory needs to say more about how urban agglomeration benefits firms of different types and sizes, and small business and self-employment research needs to say more about the influence of location, in particular cities. How businesses use both commercial and residential property are integral to the nature of growth in cities.

## 摘要

在发展中国家，小微企业（10 名以下员工）和居家企业遭到了城市经济发展研究的系统忽视。本文基于对英国长期繁荣期中四年期的一组企业层面数据的实证分析，评估了城市中座落地点以及在企业主家中经营对小微企业成长的影响。这项分析显示，城市有助于小微企业的营收增长，但无助于员工增长，表明小微企业由城市引起的额外增长可能是不增加工作岗位的增长。但是，对于居家型小微企业而言，城市促进其成长为中型企业（50 名以上员工）。结论是，包括居家企业在内的小微企业对于城市经济的发展和活力不可或缺，对于理解城市中增长的性质至关重要。集聚理论需要更多地探讨城市集聚如何让不同类型和规模的企业受益，而关于小企业和自雇型企业的研究需要更多探讨座落地点的影响，尤其是城市的影响。企业利用商业房产和居住房产的方式，是城市中增长性质不可或缺的一部分。

## Introduction

Businesses that employ no or few staff are a significant and growing part of developed economies (European Commission, 2015). Growth is particularly strong in knowledge-intensive and creative sectors (Anyadike-Danes et al., 2015) that have a strong presence in cities (D’Arcy and Gardiner, 2014). However, there is little existing research on this type of business, particularly in cities.^1^

This article is concerned with the influence cities have in assisting (or hindering) existing microbusinesses to grow. Its objective is to identify the influence a city location has on establishment-level growth among microbusinesses. In addressing this objective, the article focuses on two questions. First, whether a city location is associated with greater growth among microbusinesses. Second, whether microbusinesses based in the owners’ homes are associated with lower growth, and how this is mediated by being located in a city.

The growing number of microbusinesses reflects a set of interrelated processes, including: expansion of Information and Communication Technology (ICT) capacity and take-up; the growth of knowledge-intensive sectors; labour-market deregulation; changes to industrial organisation, including out-sourcing and project-based working; the emergence of portfolio careers; and ‘downshifting’ and lifestyle changes (Sayers, 2010). Many businesses are run from business owners’ homes, and are often referred to as home-based businesses (HBBs) (Mason et al., 2011).

Microbusinesses represent a substantial proportion of total economic activity. They accounted for 92.7% of all businesses in the EU28 non-financial business sector in 2014/2015, and most of these microbusinesses do not employ any staff (European Commission 2015: 8). Business population figures from the UK government indicate that, in 2013, 95.6% of all businesses in the UK employed fewer than 10 staff, accounting for around one third of UK employment and just below one fifth of business turnover (Department for Business, Innovation & Skills, 2014). Businesses employing fewer than 10 staff grew in number in the UK by 55 per cent between 2000 and 2013, compared to an 18 per cent increase in SMEs (those employing 10–249 staff) and a five per cent decline in the number of large enterprises with 250 or more employees (Department for Business, Innovation & Skills, 2014). These are consistent trends not obviously affected by the global financial crisis.

Microbusinesses are thought to be distinctive in a number of respects that serve to limit their potential for growth (Anyadike-Danes et al., 2015; Davidsson et al., 2006; O’Farrell and Hitchens, 1988; Penrose, 1995). First, they suffer disproportionately from ‘credit rationing’ by financial institutions. Second, a number of legal and psychological thresholds often need to be crossed on the journey out of micro status, including: registration for tax purposes; taking on employees; moving into bigger premises; or becoming an exporter. Third, microbusinesses are often characterised as economically unimportant, run by those unable to find a job as a paid employee or as ‘hobby’ businesses. Finally, it has been argued that microbusinesses run from the owner’s home (accounting for around half of all microbusinesses) lead to ‘jobless growth’ – expanding turnover but not taking on employees (Mason et al., 2011).

Remarkably, little is known about microbusinesses in developed economies, not least because many are excluded from available administrative data because they are not registered businesses due to not having employees or not being liable for tax. In particular, no systematic quantitative analysis exists (that we are aware of) on the circumstances in which non-employing microbusinesses take on employees.

There have long been calls for studies of small-firm growth to incorporate the influence of location (Mason and Harrison, 1985) or environment (Penrose, 1995) because ‘small firms are much more dependent upon external factors operating in their local milieu than are large corporations’ (O’Farrell and Hitchens, 1988: 1380). This knowledge gap has emerged due to urban economic theory tending to overlook firm size or implicitly emphasise the role of large firms (Potter and Moore, 2000).

Against this background, this article seeks to make a novel contribution to knowledge of business growth and city policies in four regards. First, it focuses on microbusinesses in cities, which have been neglected in existing research. Second, it uses a dataset that includes unregistered businesses and those which do not employ any staff. Third, it uses unique longitudinal data to measure ‘real’ growth instead of relying on business owners’ perception of growth. Fourth, it addresses the neglected transitions of becoming an employer and crossing the threshold at which tax is payable on sales, both of which are linked to a business entering datasets based on business registers. Currently little is known about businesses under these radars, let alone the factors associated with them moving above the radar.

## Business growth and the urban environment

This section discusses relevant concepts and evidence from existing literatures on: agglomeration economies; SMEs and microbusinesses; and HBBs. It highlights the empirical gap that exists at the interface between these literatures.

### Agglomeration economies and firm size

Cities are thought to offer ‘agglomeration economies’ or ‘external economies of scale’ arising from access to infrastructure, specialist labour, suppliers, customers and business networks (Duranton and Puga, 2000; Glaeser and Gottlieb, 2009; Storper and Venables, 2004). Such agglomeration benefits have been linked to enhanced business performance across a range of domains, including growth. It has been argued that large firms can internalise some aspects of agglomeration economies (for example specialist skills and training) to a greater extent than small firms (Harrison et al., 1996). This implies that microbusinesses stand to benefit more than large firms from agglomeration economies in cities, consistent with evidence on the importance of diverse labour markets and deep labour pools stated by small firms (Friedman, 1995; Leibovitz, 2004; Michimi and Berentsen, 2008). Large ‘anchor’ organisations on which small firms often rely (Leibovitz, 2004) are found mainly in conurbations and larger cities, meaning agglomeration benefits accruing to small firms may increase with city size. Yet firm size has not featured to any significant extent in analysis of agglomeration benefits and city economies.

Businesses may be able to take advantage of some of the benefits of agglomeration economies from locating in a town close to a city, but avoid the high rents and congestion in the city itself (Dijkstra et al., 2013). Technological change has enabled this locational strategy to be pursued more widely, and trade and commuting flows between cities and their wider regions are substantial (Overman et al., 2010). Nevertheless, many businesses do not relocate frequently and face constraints in where they can locate (e.g. due to business owners’ family ties and partners’ workplaces), and businesses are influenced by the urban and economic environment in which they operate, irrespective of the degree of choice they exercised in being located there.

### SMEs and microbusiness research

Measuring growth by establishment size is complex due to the dynamics of the birth, growth, shrinkage and death of businesses (Davidsson et al., 2006). By definition, a microbusiness with sustained growth will grow out of its micro status. The dynamics of firm growth, however, are not well understood and calls have been made to track growth trajectories of firms over time (Anyadike-Danes et al., 2015; Davidsson et al., 2006). At the national level in the UK, the majority of private sector employment growth over the period 1998–2013 came from firms employing fewer than 10 people (Anyadike-Danes et al., 2015). It is striking that over a similar period, the number of self-employed people in the UK increased by over half a million between 2010 and 2015 (Department for Business, Innovation & Skills, 2016). Despite these parallel trends, business research has been reluctant to incorporate self-employment. While some of the rise in self-employment is likely to be driven by precariousness in, and exclusion from, the labour market for employees, increases in self-employment are greatest in skilled service industries – sectors that are associated with growth in city economies, including education, personal and business services and media (D’Arcy and Gardiner, 2014).

Microbusinesses are often seen as non-‘mainstream’, motivated by owners’ personal interests rather than by commercial incentives, offering little potential for growth and being of more value in promoting social inclusion (Oughton et al., 2003; Servon, 1997). Sole proprietorships do indeed display lower growth than partnerships (Davidsson et al., 2006).

In Europe, the rural studies literature stresses the role of microbusinesses in promoting diversity, resilience and ability to adapt to change rather than in contributing to overall economic growth (North and Smallbone, 1996; Steiner and Atterton, 2014). A specific type of micro entrepreneur identified by rural studies, particularly in Europe, is the ‘lifestyle entrepreneur’, linked to processes of ‘downshifting’ out of pressured jobs into ways of making a living better matched to personal preferences and values, which often involves a well-educated professional moving from a city to a rural area for lifestyle reasons and to run a small business (Herslund, 2012). In addition to the ‘lifestyle entrepreneur’, entrepreneurship literature has identified the ‘creative entrepreneur’ operating in more innovative ways and in higher-tech and more knowledge-intensive industries more likely to be found in cities (Lee et al., 2004).

### Home-based businesses

Little existing business research considers the type of business premises used. This is potentially an important distinction, as many microbusinesses are run from people’s homes rather than commercial premises. Home-based businesses (HBBs) comprise the self-employed and owner managers who work from home or use the home as base for their business. Academic interest in HBBs, as with microbusinesses more generally, in developed countries has often been linked with rural economies (Newbery and Bosworth, 2010). This is why little is known about HBBs in urban areas or cities. Mason et al. (2011) found that HBBs in the UK are more prevalent in rural areas. Nevertheless, there are plenty of HBBs in cities, yet these have received little research attention (Jain and Courvisanos, 2013; Sayers, 2010). Yet it is plausible that, particularly in the creative industries and advanced business services, which are concentrated in cities, entrepreneurs often do not need commercial premises for their business as services can be provided via the internet or at the customers’ premises.

A portion of agglomeration benefits gets captured in higher land and commercial property prices in cities (Rosenthal and Strange, 2004). It could therefore be expected that HBBs may stand to benefit more from a city location by avoiding high commercial rents but at the same time benefiting from agglomeration externalities.

Existing research provides insights into the motivations for running a business from home, emphasising aspects of work-life balance (Myriel and Daly, 2009). There is little research on the growth of HBBs and thus little is known about growth strategies and relocation of HBBs that started in the owner’s home. Walker (2003) challenged the view that HBBs are mostly ‘hobby’ businesses in rural economies using a survey of businesses in Australia and concluded that HBBs generate employment. Mason et al. (2011), however, argued based on a sample of members of the Federation of Small Businesses in the UK that running a business from home generally leads to ‘jobless growth’ linked to expanding sales but not taking on employees.

### Situating our approach

Microbusinesses have been overlooked in urban economic research, yet there are good reasons to assume that they would benefit from the agglomeration economies offered by cities. Furthermore, it has been posited (although not widely investigated) that microbusinesses are more susceptible than larger firms to their local economic and built environment. Similarly, the small business literature has not considered location, including the possible role of cities, in influencing business growth.

Microbusinesses have risen in number in recent years. Self-employment and freelancing in knowledge-intensive and creative industries have risen particularly sharply (related to changes in the way large organisations use labour). These sectors are disproportionately found in cities and lend themselves to working from home. Running a business from home could be expected to bring a greater competitive advantage in cities because it allows expensive commercial rents to be avoided.

Microbusinesses are often characterised as economically unimportant and offering little potential for growth. In relation to HBBs, empirical findings are contradictory, stressing that these are ‘proper’ businesses with employment growth, on the one hand, and that they achieve ‘jobless growth’ because of physical limits to taking on employees, on the other hand. Yet businesses can move into commercial premises or take on employees who work elsewhere, so this contradiction requires further investigation using longitudinal data.

Our analysis is concerned with whether a city location is connected to the nature and extent of growth in the size of existing establishments. Establishment size has a number of dimensions. Two of the most measurable and meaningful are number of employees and turnover (Davidsson et al., 2006). Our analysis is firstly concerned with growth in number of employees for three reasons. First, cities have been identified as potential sources of job growth, particularly in the UK, but the contribution of microbusinesses to this is currently unknown. Second, micro status is conventionally defined as fewer than 10 employees, thus focusing on the number of employees enables us to identify businesses that grow out of their micro status. Third, becoming an employer is a significant step in the evolutionary growth of a business, and again focusing on number of employees allows us to identify microbusinesses that become an employer (i.e. going from zero to at least one employee).

Another significant step in the growth of an establishment is registering to pay value-added tax (VAT) on traded goods and services, which (in the UK) is only required above a certain level of turnover. Many businesses under the VAT threshold are unregistered (as long as they do not employ anybody) and thus excluded from much existing research. Therefore, our analysis secondly includes turnover growth, and captures growth over this tax threshold.

## Methods and data

### Data

Panel data are crucial in growth studies for three reasons, articulated by Davidsson et al. (2006). First, to accurately measure growth at the level of the establishment. Second, to accurately capture conditions preceding growth rather than relying on retrospective recall or self-assessed anticipated growth which are subject to reporting and selection biases. Third, to better disentangle the causality between business characteristics and growth.

This article draws on panel data from the UK Survey of Small- and Medium-sized Enterprises’ Finances (UKSSMEF).^2^ What makes these data so valuable is their panel design and the fact that registered and unregistered businesses are included.

The UKSSMEF offers the unique opportunity to follow microbusinesses over four years. A total of 2500 private sector SMEs (i.e. businesses with 0−249 employees) in the UK were interviewed in 2004. A total of 1253 of these businesses were interviewed again in 2008. In 2008 businesses that had now more than 249 employees were included because they had grown during the four-year period. For more information on sampling strategy in the UKSSMEF, see Fraser (2005, 2009).

This study measures growth directly using a longitudinal design, therefore the sample is restricted to businesses that were interviewed twice. Businesses only interviewed once (i.e. that could not be interviewed again in 2008) include both those that ceased trading and those that grew (e.g. because they moved premises) as well as others that refused to take part in the follow-up survey, making it impossible to identify businesses that ceased trading (see ‘sample attrition’ section for fuller details of the nature of attrition from the panel and its implications). In order to assess the growth of microbusinesses, 943 businesses were included in the analysis that were microbusinesses in the 2004 survey, were re-interviewed in 2008, were not part of the ethnic boost sample^3^ and for which information on number of employees and location was available. We refer to this as our ‘linked sample’, which meets the requirement of our longitudinal analysis. Of these 943 businesses in our linked sample, 139 were located in a city in 2004. In this study, microbusinesses are defined as businesses of any legal status that had fewer than 10 staff (including the owner/owner manager) in 2004.

### Models and measurement

Five measures of business growth were derived for this study – becoming an employer, growth and fast growth in employment, and growth (over the VAT threshold) and fast growth in turnover. First, businesses that had no employees in 2004 and had one or more employee in 2008 were classified as having become an employer. Second, based on employment numbers it is measured whether microbusinesses grew out of their micro status, i.e. had at most nine staff in 2004 and 10 or more staff in 2008. Third, fast job creation microbusinesses were identified that had 50 staff and more in 2008, i.e. grew from micro to medium-sized businesses. Fourth, based on turnover, businesses were identified that moved from below to above £100,000 per annum (chosen because it is the range break in the data closest to the VAT registration level, which was £77,000 until mid-2014).^4^ Fifth, microbusinesses with less than £100,000 turnover in 2004 and a turnover of £250,000 and more in 2008 were classified as high growth turnover microbusinesses. Businesses displaying high growth are sub-groups of businesses displaying growth. In other words, because our sample starts with businesses initially employing fewer than 10 staff, businesses growing to 50+ staff also grew out of their micro status. Similarly, because our turnover analysis only includes businesses initially with turnover below £100,000, businesses growing turnover to £250,000 or more by definition also moved over the £100,000 threshold.

Separate logistic regression models are specified for each of these five growth measures. The dependent variables are one if the business had grown and zero if it had, for the relevant model: remained a non-employer; remained micro; or remained below £100,000 sales turnover between the two survey years (2004 and 2008).

All explanatory variables in the growth models are measured at 2004 in order to estimate effects of conditions preceding growth. The independent variable of interest is city location, captured as a categorical variable. The location of the business draws on the self-assessment of the business owner based on a choice from four types of location: i) major conurbation; ii) city; iii) town; iv) village or rural. There are many missing responses to the turnover question in the UKSSMEF. The reduced sample size available for the turnover growth models necessitated the use of a binary dummy variable for city location (city = 1; otherwise = 0). In the employment growth models, however, the four-fold location categories are used. A more nuanced classification of urban environments and the urban system unfortunately is not available in the UKSSMEF.

Microbusinesses in cities and their owners display distinct characteristics (Supplementary Table S1) that need to be taken into account in the growth models. In particular, microbusinesses in cities show great industrial diversity; owners of microbusinesses in cities are more likely to have a university degree than owners of microbusinesses outside cities; and HBBs are less likely to be found in cities than outside cities. The relevance of a city location for microbusiness growth therefore has to be investigated as a function of these factors. The effect of city location on HBB growth is further investigated by an interaction effect between a city location and running an HBB (as opposed to commercial premises). Further control variables are: gender and age of the (principal) owner, legal status of business, receipt of business advice, whether a recently founded business and whether located in London, all of which feature in existing literature as factors influencing growth.

### Sample attrition

Of the 2500 businesses interviewed in 2004, almost exactly half (1253) were interviewed again in 2008. Business survival rates calculated using the UK’s Inter-Departmental Business Register (IDBR) are much higher than ‘survival’ to the follow-up interview in the UKSSMEF,^5^ which means that attrition in the UKSSMEF is not only due to businesses that cease trading but also because businesses could not be re-contacted (presumably including those with growth) or refused to take part in the survey for a second time. This is why we cannot identify ceased businesses in the UKSSMEF and therefore using businesses that leave the 2004 sample as a proxy for ceased trading would lead to serious biases.

Given the objective of this study, the research design is to assess the effect of being located in a city on the probability of a business growing. Our research design compares businesses that grow with businesses that do not grow, with particular reference to the influence of whether they are located in a city and the type of premises. Although microbusinesses that cease trading cannot be identified in the data, our research design does not depend on their inclusion. Because we are examining the influence of location on microbusiness growth, attrition bias would only be a problem for this study if sample attrition were stratified by location. Our assessment of attrition indicates that attrition is not stratified by location.

To test attrition bias of the sample, the group of microbusinesses in the 2004 survey that was not followed up four years later was compared with the group of follow-up microbusinesses. A logistic regression model was used for the comparison analysis. Results are available in Supplementary Table S2. However, a second interview is more likely for businesses that were an HBB in 2004. This would only be a problem for the present study if the growth of (former) HBBs in the linked sample was not equally distributed across locations. This cannot, however, be tested because we do not know if businesses leaving the sample grew.

The period between 2004 and 2008 represents a time of economic growth in the UK which explains partly why the majority of microbusinesses in the linked sample for longitudinal analysis grew (see sample description in Appendix Table A1). Our findings are thus specific to a period of economic growth.

## Results

### Descriptive analysis

Before presenting findings from multivariate growth models, this section presents descriptive results. In the linked sample, a greater proportion of microbusinesses in cities display growth than microbusinesses in other locations. This is true of both employment (Table 1) and turnover (Table 2).

**Table 1.** Microbusinesses by employment growth measures and type of location, column percentages.

Employment in 2004 and 2008	Type of location				Total
	Major conurbation	City	Town	Village/rural area	
Fewer than 10 staff in 2004 and 10+ staff in 2008	57.0	55.4	46.9	50.4	50.4
Fewer than 10 staff in 2004 and 50+ staff in 2008	15.2	20.9	15.9	13.1	15.5
Fewer than 10 staff in 2004 and 2008	43.0	44.6	53.1	49.6	49.6
Became employer (no staff in 2004 & 1+ staff in 2008)	91.1	87.3	72.4	73.2	76.7
N (< 10 in 2004)	79	139	358	367	943
N (no staff in 2004)	45	63	181	149	438

*Notes*: UKSSMEF 2004 and 2008, unweighted data.

*Source*: Authors’ compilation.

**Table 2.** Microbusinesses with < 10 staff and turnover below £100,000 in 2004 by turnover growth measures and type of location, column percentages.

Turnover in 2004 and 2008	Type of location				Total
	Major conurbation	City	Town	Village/rural area	
Below £100,000 in 2004 & £100,000+ in 2008	69.4	86.3	67.4	72.2	72.4
Below £100,000 in 2004 & £250,000+ in 2008	63.9	71.2	57.6	58.2	60.6
Below £100,000 in 2004 & 2008	30.6	13.7	32.6	27.9	27.6
N	36	73	172	158	439

*Notes*: UKSSMEF 2004 and 2008, unweighted data; businesses with a turnover of over £100,000 in 2004 are not displayed because they are not included in the subsequent modelling.

*Source*: Authors’ compilation.

As might be expected, a smaller proportion of HBBs than non-HBBs display employment growth (Table 3). In contrast, and more surprisingly, a larger proportion of HBBs than non-HBBs display turnover growth.

**Table 3.** Growth measures by city location and home-based business (HBB), in percent.

Growth measures (2004−2008)	HBB			Non-HBB		
	City	Outside city	total	City	Outside city	Total
Fewer than 10 staff in 2004 and 10+ staff in 2008	50.0	42.4	43.3	58.8	56.2	56.7
Fewer than 10 staff in 2004 and 50+ staff in 2008	20.4	9.4	10.7	21.1	19.6	19.8
Became employer (no staff in 2004 & 1+ staff in 2008)	90.6	74.0	76.1	83.9	76.3	77.5
Below £100,000 in 2004 & £100,000+ in 2008	82.5	47.5	51.9	43.4	39.6	40.3
Below £100,000 in 2004 & £250,000+ in 2008	62.5	37.9	41.0	39.1	35.0	35.8
N employment growth	54	394	448	85	409	494
N non-employment business growth	32	219	251	31	156	187
N turnover growth	40	282	322	69	303	372

*Notes*: UKSSMEF 2004 and 2008, unweighted data.

*Source*: Authors’ compilation.

Nevertheless, HBBs are no slouches in terms of jobs creation. Not far off half of micro HBBs in the linked sample grew out of their micro status between 2004 and 2008, while a sizeable proportion (11%) employed 50+ staff four years later. Furthermore, HBBs and non-HBBs without employees are equally likely to make the transition from being a non-employer to an employer.

The growth premium of being located in a city (Table 1) appears to be relatively modest for microbusinesses in commercial premises, but substantial for HBBs (Table 3). In the linked sample, HBBs in cities display growth more often than HBBs outside of cities with respect to all measures of employment and turnover growth (Table 3). Furthermore, the gap in turnover growth between HBBs and non-HBBs widens substantially in cities, where almost double the proportion of HBBs make the transition over the £100,000 threshold (83% of HBBs versus 43% of non-HBBs). Similarly, the HBB penalty on employment growth is much reduced in cities – and is reversed for becoming an employer, with 91% of sampled micro HBBs in cities becoming an employer between 2004 and 2008, versus 84% of non-HBBs.

The message from the descriptive analysis which will be tested further is that HBBs benefit more from agglomeration economies than non-HBBs. Although HBBs are disproportionately found in rural areas (in the linked sample 12% of all HBBs are found in cities compared to 17% of non-HBBs), they nevertheless represent 39% of all microbusinesses in cities in the linked sample (full locational breakdowns of HBBs and non-HBBs are available in Supplementary Tables S3 and S4).

### Modelling results

The descriptive results provide important information on the prevalence and patterns of microbusiness growth by location and type of premises. This section further explores these patterns of growth while now accounting for other characteristics that influence business growth. Table 4 displays model results for becoming an employer. Table 5 displays model results for growing out of micro status, while Table 6 reports high employment growth. Table 7 displays modelling results for turnover growth and high turnover growth.

**Table 4.** Employment growth: Non-employing businesses became employers (Group 1) versus remained non-employing business (Group 0) 2004−2008, odds ratios.

Independent variables (2004)	Model 1 (without interaction term)		Model 2 (with interaction term)	
*(ref cat = reference category)*	OR	SE	OR	SE
Location (ref cat: city)				
Major conurbation	1.225	0.944	–	–
Town	0.351**	0.177	–	–
Village/rural area	0.506	0.261	–	–
London (ref cat: outside London)	0.498	0.280	0.716	0.359
City (ref cat: not in city)	–		0.600*	0.174
Home-based business (ref cat: commercial premises)	0.869	0.281	0.761	0.249
Home-based business*City location^1^	–		2.390	1.758
Industry (ref cat: wholesale & retail)^2^				
Construction	0.195***	0.073	0.197***	0.073
Hotel and restaurant	1.134	0.680	1.092	0.655
Manufacturing	0.081***	0.035	0.081**	0.035
Sole proprietor (ref cat: not sole prop.)	0.487	0.305		
Degree (ref cat: no degree)	0.977	0.455	1.060	0.490
Age of (principal) owner	1.010	0.015	1.011	0.015
Female (principal) owner (ref cat: male)	1.922*	0.738	1.966*	0.754
Start-up business (ref cat: trading 2+yrs)^3^	0.489	0.361	0.479	0.353
External advice not received (ref cat: advice received)	0.525**	0.153	0.517**	0.151
N	409		409	
Log likelihood	−158.208		−158.834	
LR chi^2^(df)	134.00(15)		132.75(14)	
Pseudo R^2^	0.30		0.29	

*Notes*: UKSSMEF 2004 and 2008, unweighted data, independent variables are based on 2004 survey, dependent variable is derived from measuring growth between the 2004 and 2008 surveys. ^1^The interaction effect is the ratio of the odds of city and HBB and not the odds ratio as is the case for main effects. ^2^Industries with large SEs (due to few observations) are not displayed. ^3^Trading for less than 24 months. Significance: *** = 1%, ** = 5%, * = 10%.

*Source*: Authors’ compilation.

**Table 5.** Employment growth: Businesses that grew out of microbusinesses versus businesses that remained micro (< 10 staff) 2004−2008, odds ratios.

Independent variables (2004)	Model 1 (without interaction term)		Model 2 (with interaction term)	
*(ref cat = reference category)*	OR	SE	OR	SE
Location (ref cat: city)				
Major conurbation	0.959	0.364	–	–
Town	0.837	0.217	–	–
Village/rural area	1.038	0.276	–	–
London (ref cat: outside London)	0.887	0.304	0.887	0.276
City (ref cat: out of city)	–	–	0.853	0.271
Home-based business (ref cat: commercial premises)	0.509***	0.093	0.477***	0.094
Home-based business*City location^1^	–	–	1.687	0.807
Industry (ref cat: wholesale & retail)^2^				
Construction	2.998***	1.041	3.126***	1.090
Hotel and restaurant	0.837	0.366	0.864	0.376
Manufacturing	1.392	0.563	1.426	0.577
Real estate, renting and business activities	6.608***	2.292	6.645***	2.300
Transport, storage and communication	1.217	0.563	1.315	0.604
Sole proprietor (ref cat: not sole proprietor)	0.211***	0.039	0.212***	0.039
Degree (ref cat: no degree)	0.786	0.189	0.822	0.198
Age of (principal) owner	1.007	0.008	1.007	0.008
Female (principal) owner (ref cat: male)	1.189	0.252	1.201	0.254
Start-up business (ref cat: trading 2+ yrs)^3^	0.935	0.291	0.944	0.296
External advice not received (ref cat: advice received)	1.087	0.191	1.088	0.192
N	886		886	
Log likelihood	−445.726		−445.719	
LR chi^2^(df)	336.73(19)***		336.75(18)	
Pseudo R^2^	0.274		0.274	

*Notes*: UKSSMEF 2004 and 2008, unweighted data, independent variables are based on 2004 survey, dependent variable is derived from measuring growth between the 2004 and 2008 surveys. ^1^The interaction effect is the ratio of the odds of city and HBB and not the odds ratio as is the case for main effects. ^2^Industries with large SEs (due to few observations) are not displayed. ^3^Trading for less than 24 months. Significance: *** = 1%, ** = 5%, * = 10%.

*Source*: Authors’ compilation.

**Table 6.** Fast employment growth: Businesses that were micro in 2004 (< 10 staff) and had 50 or more staff in 2008, odds ratios.

Independent variables (2004)	Model 1 (without interaction term)		Model 2 (with interaction term)	
*(ref cat = reference category)*	OR	SE	OR	SE
Location (ref cat: city)				
Major conurbation	0.635	0.286	–	–
Town	0.857	0.263	–	–
Village/rural area	0.997	0.328	–	–
London (ref cat: outside London)	0.899	0.383	0.804	0.320
City (ref cat: out of city)	–	–	0.767	0.274
Home-based business (ref cat: commercial premises)	0.635**	0.148	0.526**	0.136
Home-based business* City location ^1^	–	–	3.134**	1.790
Industry (ref cat: wholesale & retail)^2^				
Construction	0.956	0.677	0.965	0.685
Hotel and restaurant	0.685	0.650	0.682	0.647
Manufacturing	2.103	1.564	2.183	1.626
Transport, storage and communication	1.114	1.057	1.211	1.145
Sole proprietor (ref cat: not sole proprietor)	0.232***	0.057	0.226***	0.055
Degree (ref cat: no degree)	0.810	0.224	0.780	0.221
Age of (principal) owner	0.998	0.010	0.999	0.010
Female (principal) owner (ref cat: male)	0.659	0.177	0.677	0.181
Start-up business (ref cat: trading 2+yrs)^3^	0.652	0.229	0.684	0.240
External advice not received (ref cat: advice received)	0.907	0.213	0.919	0.215
N	886		886	
Log likelihood	−292.545		−291.131	
LR chi^2^	178.03(19)***		180.86(18)***	
Pseudo R^2^	0.233		0.237	

*Notes*: UKSSMEF 2004 and 2008, unweighted data, independent variables are based on 2004 survey, dependent variable is derived from pooling the 2004 and 2008 surveys. ^1^The interaction effect is the ratio of the odds of city and HBB and not the odds ratio as is the case for main effects. ^2^Industries with large SEs (due to few observations) are not displayed. ^3^Trading for less than 24 months. Significance: *** = 1%, ** = 5%, * = 10%.

*Source*: Authors’ compilation.

**Table 7.** Microbusinesses with turnover increase above £100,000 (Model 1) and £250,000 (Model 2) versus those that remained below £100,000 turnover 2004−2008, odds ratios.

Independent variables (2004)	Model 1 (£100,000)		Model 2 (£250,000)	
*(ref cat = reference category)*	OR	SE	OR	SE
City (ref cat: out of a city)	3.407***	1.440	2.366**	0.810
Degree (ref cat: no degree)	0.639	0.229	0.572*	0.188
Age of (principal) owner	1.021*	0.0128	1.015	0.012
Female (principal) owner (ref cat: male)	1.157	0.381	1.518	0.463
London (ref cat: outside London)	0.583	0.245	0.837	0.341
Home-based business (ref cat: commercial premises)	0.981	0.279	0.787	0.204
Sole proprietor (ref cat: not sole proprietor)	0.193***	0.079	0.318***	0.104
Industry (ref cat: wholesale & retail)^1^				
Construction	0.077***	0.031	0.066***	0.025
Hotel and restaurant	0.437	0.227	0.122***	0.053
Manufacturing	0.059***	0.029	0.036***	0.019
Real estate, renting and business activities	0.408**	0.161	0.371***	0.129
Transport, storage and communication	0.476	0.260	0.227***	0.107
Start-up business (ref cat: trading 2+ yrs)^2^	0.737	0.491	0.690	0.434
External advice not received (ref cat: advice received)	0.924	0.249	0.710	0.180
N	418		418	
Log likelihood	−191.413		−212.566	
LR chi^2^(14)	112.81***		137.34***	
Pseudo R^2^	0.228		0.244	

*Notes*: UKSSMEF 2004 and 2008, unweighted data, independent variables are based on 2004 survey, dependent variables are derived from pooling the 2004 and 2008 surveys. ^1^All other industries omitted from models because of ‘empty cells’. ^2^ Trading for less than 24 months. Significance: *** = 1%, ** = 5%, * = 10%.

*Source*: Authors’ compilation.

In Tables 4–6, two models are displayed in which Model 2 adds an interaction term between HBB and a city location to Model 1 in order to test whether HBBs in a city are more likely to grow. The interaction term tests whether being an HBB in a city has a different effect on employment growth or turnover growth than being an HBB outside of a city. In order for the interaction term to solely capture the effect of cities on HBBs, it is necessary to keep the main effects (of being an HBB and being in a city) in the models.

### Employment growth

In the descriptive analysis, cities display higher proportions than towns or villages/rural areas of microbusinesses that became an employer, grew out of their micro status or increased their staff substantially to 50 or more (Table 1). However, the numerically higher employment growth in cities appears to be due mainly to industry effects. Once industrial composition is controlled for in logistic regressions of these three measures of employment growth/non-growth (Tables 4–6), the effect of a city location disappears or is largely reduced. The only exception is becoming an employer, which is (still) higher amongst microbusinesses in cities than amongst those in towns (Model 1, Table 4).

One explanation for this is that the agricultural sector is more likely to display employment growth in the microbusiness sector. Another explanation is that industries that are mainly based in cities are also more likely to grow in terms of employment, for example real estate, renting und business activities (Table 5). Microbusinesses in construction and manufacturing are much less likely to become employers (Table 4), although construction businesses are more likely to grow out of their micro status (Table 5).

Sole proprietorships are substantially less likely to grow out of their micro status (Table 5) or to display high employment growth (Table 6). However, sole proprietors are as likely to become an employer compared to other legal forms of microbusiness, meaning they are more likely to remain microbusinesses (Table 4).

HBBs were, unsurprisingly, statistically significantly less likely to grow out of their micro status or display high employment growth than businesses in commercial premises (Model 1 in Tables 5 and 6). However, HBBs were as likely as microbusinesses in business premises to become an employer, even after controlling for industry and legal status^6^ (Model 1, Table 4), suggesting that they do grow but often remain a microbusiness. These results indicate that HBBs are more likely to grow in cities than outside cities, independent of their legal status.

The descriptive statistics suggested that HBBs behave differently in cities. This is confirmed in the multivariate models (Model 2 in Tables 4–6). HBBs are substantially and significantly *more* likely to display high employment growth when they were located *in cities* after controlling for industry and other factors (Table 6).^7^ The HBB variable now indicates the effect of being an HBB on fast employment growth only for HBBs located outside of a city. Thus, outside cities the odds of fast employment growth are reduced by half for HBBs compared to businesses in commercial premises. In cities, the odds of fast employment growth is 1.65 for HBBs compared to 0.6 for businesses in commercial premises.^8^ The models of becoming an employer (Table 4) and growing out of micro status (Table 5) also indicate positive effects of cities on the growth of HBBs, although the interaction terms in these models are not statistically significant. In cities, non-HBBs are less likely to become an employer than outside a city (the city dummy in Model 2 in Table 4 measures the effect for non-HBBs), although this is only significant at the 10% level and is not evident in the descriptive statistics (Table 3) so is likely to be accounted for by industry effects.

### Turnover growth

Model results indicate that microbusinesses in the linked sample in cities are substantially more likely to display turnover growth than microbusinesses outside of cities. Given that cities are not associated with greater employment growth (at least after controlling for industry and other factors), these findings indicate ‘jobless growth’ amongst microbusinesses in cities, suggesting this is not specific to HBBs (Mason et al., 2011). Microbusinesses in the linked sample that were located in cities were three times more likely to increase their turnover from below £100,000 to over £100,000 (Model 1, Table 7) and twice as likely to realise an increase to over £250,000 (Model 2, Table 7), holding other factors constant.

Sole proprietors’ turnover was most likely to remain below £100,000 in both years. HBBs in general have no effect on the turnover growth of microbusinesses. The impact on growth above £100,000 turnover is almost zero (Model 1 in Table 7).

Observations of HBBs by location and turnover growth are too few to incorporate an interaction term in Table 7 (as in Tables 4–6 on employment growth). However, the descriptive results for turnover growth displayed in Table 3 are consistent with the benefit of being located in a city specific to HBBs found previously for high employment growth.

## Conclusions

This article addressed the issue that little is known from existing literature about microbusinesses in city economies in developed countries, particularly in relation to growth. Using panel data and following microbusinesses in the UK from 2004 to 2008, this article tested whether the growth of microbusinesses in terms of employment and turnover depends on a city location and on whether run from the business owner’s home. An important empirical contribution of this article lies in the inclusion of unregistered, home-based and non-employing businesses. A longitudinal study design is crucial in revealing the lifecycle of businesses that may start in the owner’s home but subsequently move into commercial premises. Snapshots of businesses at a point in time may give a misleading picture of their growth prospects.

Findings reveal that cities provide benefits to microbusinesses for turnover growth but not for employment growth. However, in the case of microbusinesses run from the owner’s home, cities facilitate growth into medium-sized businesses (with 50+ staff), in contrast to outside of cities where HBBs have lower employment growth than businesses in commercial premises. In addition, HBBs are as likely to become an employer (shifting from having no employees to having one or more employees four years later) compared to businesses in commercial premises.

These findings make it difficult to avoid the conclusion that microbusinesses and HBBs in particular are integral to the functioning of urban economies. Microbusinesses and how they use both commercial and residential property are essential to understanding the nature of growth in cities.

An explanation is required of why cities enhance microbusiness turnover but not employment growth, which may represent jobless growth. One plausible explanation, consistent with urban agglomeration theory, is that the greater availability of local suppliers and sub-contractors in cities enables businesses with expanding sales to meet demand through buying services from sub-contractors rather than hiring new staff. Furthermore, in cities there may be greater costs associated with moving into larger premises in order to hire additional staff because land and property prices are higher in cities – examples of agglomeration diseconomies. A consequence of this sub-contracted growth interpretation is that apparent jobless growth observed at the level of the individual business is not in fact jobless at the city level – it could just be that jobs are created in other businesses used as contractors. In the case of job creation via sub-contractors, the further question arises as to the extent to which there is leakage of growth to firms located outside the city economy.

An explanation is required of the synergy that exists between being located in a city and based in the business owner’s home. One possible explanation is that compared to microbusiness owners outside of cities, the owners of microbusinesses in cities have specialist skills and networks not captured in qualifications, some of which may have been obtained when working as employees of large city-based organisations (Delmar and Davidsson, 2000), and start businesses in high-growth niches that lend themselves (at least when small) to being run from home, for example because services or products that can be delivered electronically. Face-to-face contact was found to be important for business growth particularly at the start of the business (Greve and Salaff, 2003). Another possible explanation is that (former) HBBs are more likely to be able to expand into commercial premises due to a greater diversity and availability of premises in cities.^9^ Finally, it is possible that in cities HBBs can more readily hire remote employees (people who work from their own home or at customers’ premises) because any necessary periodic face-to-face contact between the business owner and employees is made easier in a city. Engagement of small business research with research findings on self-employment and freelancing could be useful for providing more insight into the HBB and city location link discovered in this study. Future research is required to differentiate between, on the one hand, home-based self-employment driven by limited opportunities in the labour market and, on the other hand, entrepreneurial home-based self-employment which may subsequently lead to business creation and growth.

Our posited interpretations point to three specific questions for further research. First, what are the mechanisms through which a city location makes microbusiness turnover growth more likely, including the availability of customers, suppliers, business networks or links to anchor organisations? Second, what are the mechanisms by which HBBs generate employment growth – in particular is it by taking on remote employees or moving into business premises? Third, why are (formerly) home-based microbusinesses more likely to generate employment when located in cities, for example through occupying specific high-growth niches or the greater availability in cities of commercial premises to move into?

The findings reveal important heterogeneities between businesses in how they benefit from a city location, in particular in relation to whether they are run from the business owner’s home or in commercial premises. This underlines the dangers in economic models in which firms are treated as essentially homogenous (see Ottaviano, 2011).

Existing models of agglomeration externalities and urban growth tend to either not consider firm size at all or to implicitly emphasise the role of large enterprises or branches. The findings reported in this article suggest that more attention should be paid in urban studies to the role of microbusinesses in city economies (for instance as rural studies has done for family businesses). More generally, agglomeration theory needs to explain how and why agglomeration benefits and advantages for growth accrue differently to firms of different sizes. The benefits of a city location may be quite specific to certain types and sizes of business and to certain outcomes. This article has focused on the role of cities in promoting microbusiness growth. Whether the findings extend to other economic outcomes, including productivity, wages, profit and innovation, would be a fruitful area for further investigation.

Existing models of firm-level growth tend to focus on the internal characteristics of businesses and/or business owners, and not consider the influence of externalities arising from location. The findings reported in this article suggest that more attention should be paid to location, in particular how different types of urban environment and sizes of settlement across the urban system influence microbusinesses. HBBs are often somewhat dismissed in studies of enterprise growth as hobby or lifestyle businesses. The findings of this study indicate that many HBBs, particularly in cities, are serious businesses that display growth.

## Supplementary Material

Supplementary material

## Appendix

**Table A1.** Sample description.

Variable	Per cent or mean	n
Employment growth		
Became employer (no staff in 2004 & 1+ staff in 2008)	76.7%	336
Growth out of micro (10+ staff in 2008)	50.4%	475
Remained micro (< 10 staff in 2004 & 2008)	49.6%	468
Fast growing (50+ staff in 2008)^1^	15.5%	146
Turnover growth		
Below £100,000 in 2004 & above in 2008	45.6%	317
Below £100,000 in 2004 & 2008	17.4%	121
Above £100,000 in 2004 & 2008	31.1%	216
Above £100,000 in 2004 & below in 2008	5.9%	41
Below £100,000 in 2004 & above £250,000 in 2008 (fast-growing)^1^	38.1%	265
Principal owner with degree (yes)	17.8%	168
Female principal owner (yes)	26.0%	245
Age of principal owner – mean (sd)	50.3 (11.2)	927
Region		
London	7.6%	72
East	9.5%	90
East Midlands	8.6%	81
North East	8.3%	78
Northern Ireland	6.2%	58
North West	8.9%	84
Scotland	8.2%	77
South East	9.3%	88
South West	8.9%	84
Wales	8.3%	78
West Midlands	7.3%	69
Yorkshire & Humberside	8.9%	84
Home-based business (yes)	47.6%	448
Sole proprietor (yes)	65.0%	613
External advice not received (yes)	36.4%	328
Start-up business (yes)	8.7%	82
Industry		
Wholesale and retail	9.1%	86
Agriculture	7.1%	67
Construction	21.9%	206
Health and social work	7.4%	70
Hotel and restaurant	8.2%	77
Manufacturing	8.3%	78
Other community, social and personal service activities	11.8%	111
Real estate, renting and business activities	20.3%	191
Transport, storage and communication	6.0%	57

*Notes*: UKSSMEF 2004, 2008, unweighted data. N = 943 microbusinesses with fewer than 10 staff in 2004; thereof n = 438 businesses had a turnover below £100,000 in 2004. ^1^Fast-growing businesses are a subgroup of growing businesses.

*Source*: Authors’ compilation.

## Notes

1. The term ‘city’ is used in this article to refer to settlements large enough to generate non-trivial agglomeration externalities, whereas ‘urban’ is used to refer to built-up areas more generally. Conurbations and larger cities could be expected to offer greater agglomeration externalities, and qualitatively different infrastructure and sectors, than smaller cities and towns. Nevertheless, for brevity and to match categories used in the empirical analysis reported in this article (see Methods section), we restrict ourselves to the generic term ‘city’.

2. Data descriptions and Public Use Files are available through the UK Data Service (Study Numbers 5326 and 6314 for the 2004 and 2008 study respectively). Users have to be registered with the UK Data Service to download the dataset. Full information about the studies including sample design, questionnaire and sample description are included in Fraser (2005, 2009). The 2009 study is not suitable for the purpose of this article and is therefore not used.

3. In order to use a random sample as baseline, businesses that were part of the ethnic boost were excluded from the pooled sample created for this study.

4. Businesses were asked about the exact value of turnover (in the last completed accounting period) and alternatively a range could be chosen. This latter categorical information was used due to higher missing values in the other (numeric) turnover variable.

5. Four-year survival data for newly registered private enterprises (for VAT and/or PAYE) in the UK’s IDBR in 2006 was 53.2% (ONS, 2012). The ‘survival’ rate in the UKSSMEF is lower than this at 50%, but this includes unregistered businesses which could be expected to have a lower survival rate than registered businesses. Our calculations show that in the UKSSMEF only 38% of VAT-registered businesses and 39% of those with at least one employee were interviewed again in 2008. The UKSSMEF relates to a four-year period and IDBR business survival rate to a three-year period; however, given the order of magnitude of difference, it is only reasonable to conclude that sample attrition from the UKSSMEF is in excess of what would be expected due to businesses ceasing trading.

6. While there is inevitable overlap between HBBs and sole proprietors given that both are widespread in the microbusiness population, HBBs are slightly more likely to be run by a sole proprietor, and sole proprietorships are only slightly more likely to be an HBB: 68.8% of HBBs are sole proprietorships compared to 61.7% of non-HBBs; 50.2% of sole proprietors run HBBs compared to 47.6% of all microbusinesses. The model results are robust with respect to collinearity. See Supplementary Table S5 for further assessment of the relationship between HBBs and sole proprietorships.

7. The value of 3.134 for the interaction term is the ratio of the odds ratios of the included variables in the interaction.

8. Note that because odds ratios are displayed the main effect and the interaction effect have to be multiplied. The odds of fast employment growth of businesses in commercial premises in cities is 0.249; the odds of HBB in a city location is 0.411.

9. The dataset used in this study only recorded whether a business was run from home in 2004 and not in the follow-up 2008 survey, making it not possible to follow moves of HBBs into commercial premises.
