# Supplementary material for: City economies and microbusiness growth
Source: Urban Stud. 2017 Jan 11;54(14):3199–217. doi: 10.1177/0042098016680520 (PMC6187853; doi:10.1177/0042098016680520)
Supplement: Supplementary material [file USJ_680520_Supplementary_table.pdf]

Supplementary Table S1. Characteristics of microbusinesses in cities versus outside cities, 2004, odds ratios

**Independent variables**<br>*(ref cat = reference category)*<br>*('yes' in brackets indicates the category coded as 1 in binary 1/0 variables)*	**OR**	**SE**
Degree (yes)	1.737**	0.426
Female (principal) owner (yes)	0.788	0.202
Age of (principal) owner	1.014	0.010
Region (ref cat: London)		
East	0.261**	0.145
East Midlands	0.264**	0.133
North East	1.029	0.451
Northern Ireland	0.828	0.389
North West	0.623	0.266
Scotland	0.939	0.385
South East	0.386**	0.180
South West	0.430*	0.202
Wales	0.543	0.236
West Midlands	0.738	0.327
Yorkshire & Humberside	0.706	0.302
Percentage of sales exported (ref cat: nil)		
1-49%	1.118	0.404
50%+	3.174**	1.737
Home-based business (yes)	0.675*	0.157
Sole proprietor (yes)	0.727	0.168
Turnover last year <£100,000 (yes)	1.204	0.294
External advice not received (yes)	0.929	0.201
Business is 10 years and older (yes)	0.819	0.187
Industry (ref cat: Wholesale & retail)		
Agriculture	0.201**	0.163
Construction	2.108*	0.918
Health and social work	3.069**	1.538
Hotel and restaurant	3.560**	1.788
Manufacturing	2.281*	1.139
Other community, social and personal service activities	3.173***	1.389
Real estate, renting and business activities	2.289**	0.953
Transport, storage and communication	1.608	0.839
Owners net worth <£50,000 (yes)	0.842	0.220
N	945	
Log likelihood	-353.526	
LR $chi^2(30)$	85.77***	
Pseudo R^2	0.108	

Notes: UKSSMEF 2004, unweighted data
Significance: ***=1%, **=5%, *=10%
Source: authors' compilation

Supplementary Table S2. Follow-up sample (group 1) compared to drop-out (Group 0), logistic regression, odds ratios

**Independent variables** *(ref cat = reference category)* *('yes' in brackets indicates the category coded as 1 in binary 1/0 variables)*	**OR**	**SE**
Location (ref cat: city)		
Major conurbation	1.266	0.596
Town	1.195	0.407
Village/rural area	1.388	0.518
Degree (yes)	0.517**	0.164
Female (principal) owner (yes)	1.846*	0.594
Age of (principal) owner	0.992	0.012
London (yes)	0.748	0.318
No sales exported (yes)	0.879	0.322
Home-based business (yes)	5.858***	2.320
Sole proprietor (yes)	7.866***	2.129
External advice not received (yes)	1.390	0.378
Business is 15 years and older (yes)	0.577**	0.149
Industry (ref cat: Hotel & restaurant)[1]		
Construction	2.951	2.818
Health and social work	0.5980	0.589
Manufacturing	2.759	2.931
Other community, social and personal service activities	0.119***	0.095
Real estate, renting and business activities	0.390	0.314
Transport, storage and communication	0.063***	0.051
Wholesale and retail	0.094***	0.074
Owners net worth <£50,000 (yes)	1.493	0.473
N	973	
Log likelihood	-231.505	
LR $chi^2(20)$	275.52	
Pseudo R^2	0.373	

Notes: UKSSMEF 2004, unweighted data. Greater chance of attrition if Odds Ratio <1.0.

Significance: ***=1%, **=5%, *=10%

[1] Agriculture not shown.

Source: authors' compilation

Supplementary Table S3. Microbusinesses in the linked sample by HBB and location, column percentages

Location type (2004)[1]	HBB	Non-HBB
City	12.1	17.2
Major conurbation	6.9	9.7
Town	33.7	41.9
Village or rural area	47.3	31.2
N	448	494

Notes: UKSSMEF 2004 and 2008, unweighted data; only businesses that had less than 10 employees in 2004.
[1] Respondents' own assessment.
Source: authors' compilation

Supplementary Table S4. HBBs and non-HBBs as a proportion of all microbusinesses in the linked sample by location, row percentages

Location type (2004)[1]	HBB	Non-HBB	n
City	38.9	61.1	139
Major conurbation	39.2	60.8	79
Town	42.2	57.8	358
Village or rural area	57.9	42.1	366
N	448	494	942

Notes: UKSSMEF 2004 and 2008, unweighted data; only businesses that had less than 10 employees in 2004.
[1] Respondents' own assessment.
Source: authors' compilation

Supplementary Table S5. Micro home-based businesses and micro non-home-based businesses by legal status and city location in the linked sample

Characteristics in 2004[1]	%	N (sample)
Sole proprietors		
Sole proprietors - % of all microbusinesses	65.0	943
In another legal status - % of all microbusinesses	35.0	943
Sole proprietors that were in a city - % all sole proprietors	14.2	613
Microbusinesses in another legal status that were in a city - % all microbusinesses in another legal status	15.8	330
Is an HBB - % all sole proprietors	50.2	613
Is an HBB - % all sole proprietors in a city	43.7	87
Home-based business (HBB)		
Is a sole proprietor - % of all HBB	68.8	448
HBB sole proprietors that were in a city - % of HBB sole proprietors	12.3	308
HBB in another legal status that were in a city - % of HBB in another legal status	11.4	140
Non-home-based business (non-HBB)		
Is sole proprietor - % of all non-HBB	61.7	494
Non-HBB sole proprietors that were in a city - % of non-HBB sole proprietors	16.1	305
Non-HBB in another legal status that were in a city - % of non-HBB in another legal status	19.1	189

Notes: UKSSMEF 2004 and 2008; unweighted data; only businesses that had less than 10 employees in 2004.
[1] In 2004; there is no information in the UKSSMEF on type of premises and city location in the 2008 dataset.
Source: authors' compilation
